# New Long-Term Use Solid Bismuth Microelectrode Arrays for Rapid and Sensitive Determination of Sunset Yellow in Isotonic Beverages and Water Samples by Adsorptive Stripping Voltammetry

**DOI:** 10.3390/molecules30020345

**Published:** 2025-01-16

**Authors:** Mieczyslaw Korolczuk, Iwona Gęca, Artur Mazurek, Paulina Mrózek

**Affiliations:** 1 Institute of Chemical Sciences, Faculty of Chemistry, Maria Curie Sklodowska University, 20-031 Lublin, Poland; mieczyslaw.korolczuk@mail.umcs.pl (M.K.); paulina.mrozek01@wp.pl (P.M.); 2 Department of Analysis and Food Quality Assessment, Faculty of Food Science and Biotechnology, University of Life Sciences, 20-704 Lublin, Poland; artur.mazurek@up.lublin.pl

**Keywords:** Sunset Yellow, solid bismuth microelectrode array, adsorptive stripping voltammetry, determination

## Abstract

This article reports on the long-term use, solid bismuth microelectrode arrays for the first time. The presented working microelectrode is characterized by particular advantages compared to bismuth film electrodes and solid single bismuth microelectrodes; these advantages include environmentally friendly properties and the amplification of recorded currents, which are subsequently more resistant to interference. The proposed solid bismuth microelectrode array was applied to develop an adsorptive stripping voltammetric procedure for Sunset Yellow determination. The main experimental parameters were optimized. The calibration graph was linear from 5 × 10^−9^ to 1 × 10^−7^ mol L^−1^ (time of accumulation, 60 s). The detection limit was equal to 1.7 × 10^−9^ mol L^−1^. The relative standard deviation for a concentration of Sunset Yellow of 2 × 10^−8^ mol L^−1^ was 4.1% (n = 7). Potential interference effects were examined. The presented analytical procedure was applied for the determination of Sunset Yellow in isotonic beverages and the results were confirmed by HPLC as a comparative method. The correctness of the presented procedure was also confirmed by satisfactory recovery values obtained during the analysis of spiked environmental water samples.

## 1. Introduction

2–hydroxy–1–(4–sulfonatophenylazo)naphthalene–6–sulfonate, also known by its common name, Sunset Yellow FCF, is a water soluble, highly stable orange synthetic azo dye, denoted in Europe as E110. This azo dye is used as a coloring agent in many foodstuffs, e.g., confectionary and fine baked goods, powdered drinks, sweets, sports beverages, ice creams, and gelatin. As reported in [1], the daily permissible intake of Sunset Yellow FCF is equal to 4 mg kg^−1^ of body weight. This is due to the fact that the excessive consumption of Sunset Yellow FCF may manifest in a number of ways, including the development of symptoms such as hyperactivity, allergic reactions, asthma attacks, nausea, vomiting, and even immunotoxicity [2,3,4]. In consideration of the aforementioned points, it is evident that there is a necessity for the development of analytical procedures for monitoring low concentration levels of Sunset Yellow FCF in diverse food products, e.g., liquid samples as well as environmental water samples.

To date, many analytical methods have been used for the determination of Sunset Yellow in various sample types. These methods are as follows: high-performance liquid chromatography (HPLC) with UV–Vis [5,6] or mass spectrometric detection [7,8], spectrophotometry [9,10,11], and capillary electrophoresis [12,13,14].

Stripping voltammetry has also been often reported for azo dye determination. The aforementioned analytical method is of significant importance and competitiveness, as evidenced by the following advantages: relatively short measurement times, portable and cost-effective devices, possibility of conducting field analysis, and low detection limits. In stripping voltammetric measurements, the appropriate selection of a working electrode is a crucial parameter due to the limitations associated with the pH of the supporting electrolyte and the operational potential window of the electrode in question. For Sunset Yellow determination, the following working electrodes have been used: hanging mercury drop electrodes [15,16], bismuth film electrodes [17,18], glassy carbon electrodes with different surface modifications [19,20,21,22,23,24], carbon paste electrodes with various compositions [25,26,27], and boron-doped diamond electrodes [28].

A particular group of working electrodes employed in voltammetric measurements are microelectrodes. The application of microelectrodes has been demonstrated to yield specific advantages, as evidenced by many scientific publications [29,30,31]. The use of ensembles or arrays of microelectrodes offers an additional advantage in that the recorded currents are enhanced and more resistant to interference [32]. In the literature, various constructions of ensembles/arrays of microelectrodes have been reported [33,34,35,36,37].

To date, to our knowledge, there is a lack of literature on the use of microelectrodes to determine Sunset Yellow FCF via stripping voltammetry.

In the present article, a new long-term use array of solid bismuth microelectrodes is proposed. The construction method of the microelectrode in question yields several advantages, including enhanced stability, durability, and the possibility of repeated and long-term use in contrast to screen-printed electrodes [38]. Additionally, currents recorded with the use of the proposed sensor are enhanced and more stable in comparison to single solid bismuth microelectrodes [39,40]. The presented array of solid bismuth microelectrodes was utilized for the development of an analytical procedure for Sunset Yellow FCF determination through adsorptive stripping voltammetry. The developed procedure is characterized by satisfactory analytical performance. The presented procedure was used for the analysis of commercial isotonic beverages and spiked environmental samples, obtaining results consistent with those achieved using HPLC as a comparative method and satisfactory recovery values.

## 2. Results

### 2.1. Electrochemical Properties of the Solid Bismuth Microelectrode Array

**Figure 1:**
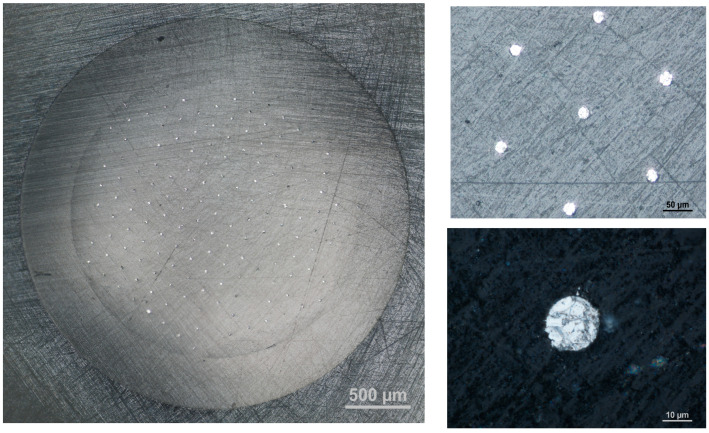
A real view of the surface of a solid bismuth microelectrode array taken by the MA200 Inverted Metallographic Microscope, depicted in various close-up views.

The presented article reports for the first time the application of a solid bismuth microelectrode array in stripping voltammetric measurements. Thanks to its construction, the proposed sensor is characterized by important advantages: (i) environmentally friendly properties, e.g., the addition of Bi(III) ions to the supporting electrolyte for bismuth film preplating is unnecessary; (ii) the amount of metallic bismuth needed for the preparation of a solid bismuth microelectrode array is drastically minimized as compared to solid bismuth electrodes of conventional sizes [41]; (iii) the recorded currents are amplified and larger as compared to a single solid bismuth microelectrode [39,40]; and (iv) with microelectrode arrays, the background current interferes to a lesser extent with the analytical signal. Real-life images of the surface of a solid bismuth microelectrode array taken with the MA200 Inverted Metallographic Microscope, depicted as various close-up views, are presented in Figure 1. 

**Figure 2:**
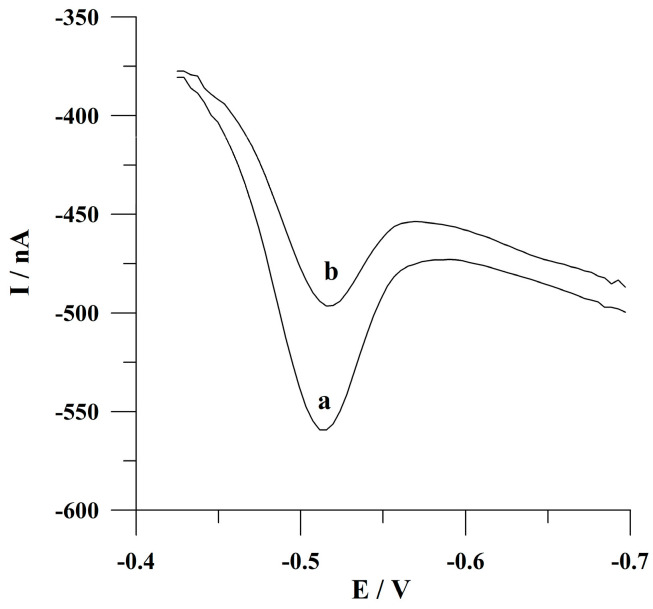
The adsorptive stripping voltammograms obtained for Sunset Yellow determination from (a) a stirred solution during a preconcentration step; (b) an unstirred solution during a preconcentration step. Potential and time of accumulation: −0.425 V, 60 s. The concentration of Sunset Yellow was 5 × 10^−8^ mol L^−1^.

The proposed working electrode was applied for the development of the analytical procedure of adsorptive stripping voltammetric determination of Sunset Yellow because preliminary experiments yielded a satisfactory analytical performance, as evidenced by the well-shaped analytical signal depicted in Figure 2. The mechanism of the process giving rise to the analytical signal of Sunset Yellow is related to its electrochemical reduction which has been clearly described and reported previously [42]. The authors demonstrated that the azo group (–N=N–) is reduced first. Shortly, the electroreduction process of Sunset Yellow consists of two stages: the first one pertains to the reversible two-electron reduction in azobenzene to hydrazobenzene; and the second stage is based on the irreversible two-electron reaction followed by breaking the azo group and leading to the formation of aromatic amino compounds.

Furthermore, Figure 2 presents the analytical responses of Sunset Yellow determination from a stirred or an unstirred solution during the preconcentration step while using the proposed solid bismuth microelectrode array. As demonstrated in Figure 2, the peak current of the analyte is approximately two times lower when a microelectrode array is employed. The received results differ significantly from those obtained using a conventionally sized solid bismuth electrode (diameter of 1 mm), where the peak current obtained from the unmixed solution was about eleven times lower as compared to the results obtained from the mixed solution in the accumulation stage. This can be explained by the fact that near the surface of a solid bismuth electrode, mass transport occurs by linear diffusion. Conversely, in the proximity of the surface of a solid bismuth microelectrode array, radial diffusion predominates, which is a way of transporting the analyte when the working electrode has microelectrode properties [43]. The present results also confirmed that, in the case of the proposed working electrode, microelectrode characteristics predominate.

### 2.2. Optimization of the pH of the Supporting Electrolyte

**Figure 3:**
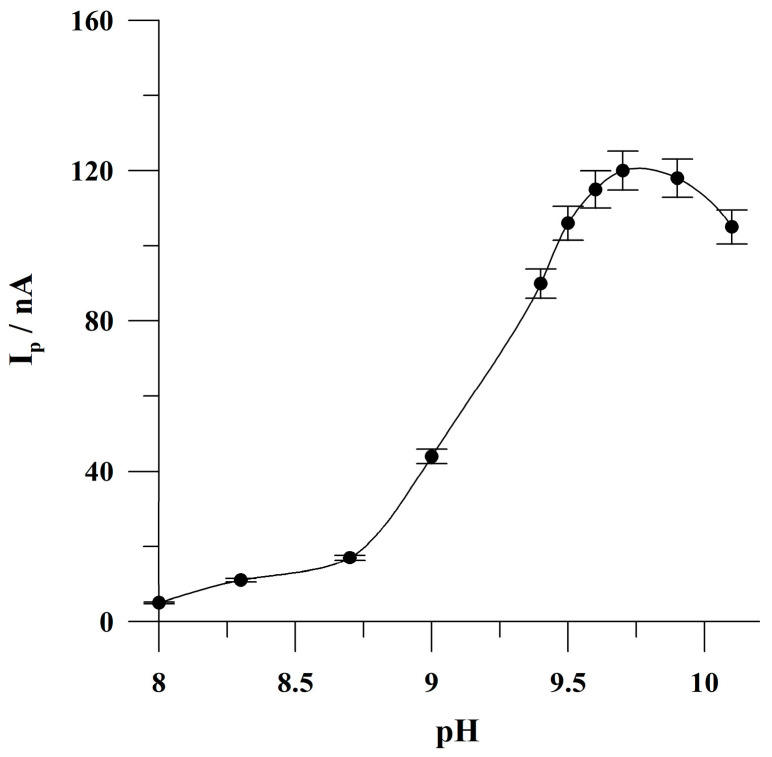
The impact of the pH of the supporting electrolyte on Sunset Yellow peak current. Concentration of Sunset Yellow: 5 × 10^−8^ mol L^−1^. Accumulation conditions: −0.425 V, 60 s. The error bars represent the standard deviation (n = 3).

The influence of the pH of the supporting electrolyte was studied in the range from 8.15 to 10.05 for a solution containing 5 × 10^−8^ mol L^−1^ of Sunset Yellow. The pH of the supporting electrolyte was adjusted by the addition of the appropriate quantity of sodium hydroxide to the supporting electrolyte which has a pH of 8.15. The obtained results are presented in Figure 3. It was observed that the peak current of Sunset Yellow increased significantly from a pH of 8.15 to 9.7, obtained the highest value at a pH of 9.7 and then slightly decreased at higher pH values. Further studies were conducted at a pH of 9.7 as an optimal value. Based on the literature data [9], it can be concluded that the unprotonated tri-anions are the main form of Sunset Yellow which underwent adsorption on the surface of the proposed microelectrode array under optimal measuring conditions.

### 2.3. Optimization of Potential and Time of the Activation Step

**Figure 4:**
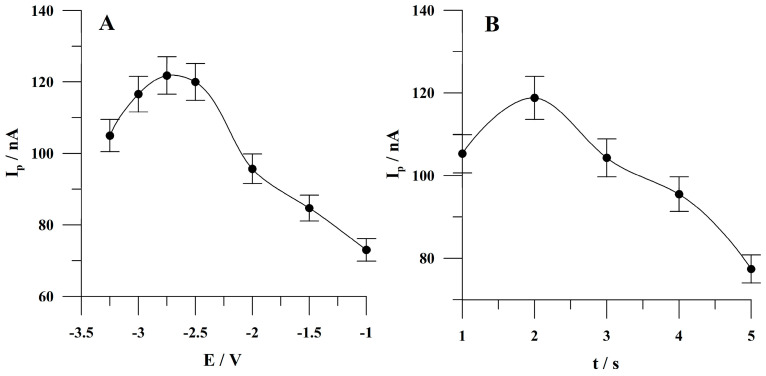
The impact of the activation potential (**A**) and activation time (**B**) on Sunset Yellow peak current. Concentration of Sunset Yellow: 5 × 10^−8^ mol L^−1^. Accumulation conditions: −0.425 V, 60 s. The error bars represent the standard deviation (n = 3).

The so-called activation step has been previously introduced to voltammetric measurements in the course of using solid metal electrodes [39,40,41] for the preparation of their surfaces before the next measurement by reduction of metal oxides to the metallic state. These oxides can potentially be formed as a result of oxidation of electrode material with oxygen present in the analyzed solution. In the present study, activation was carried out as the first step of a standard measurement procedure. In the case of the present study, the activation potential was changed in the range from −1.0 to −3.25 V. The concentration of Sunset Yellow was 5 × 10^−8^ mol L^−1^. The obtained results are shown in Figure 4A. It was observed that the peak current of Sunset Yellow increased as the activation potential was changed from −1.0 to −2.75 V and then slightly decreased at more negative potential values. Further studies were performed at the activation potential of −2.75 V as an optimal value. In such conditions, a reduction of hydrogen ions occurs; however, as was previously reported [44], accumulation conducted even at such negative potential values when using microelectrodes leads to reproducible results.

The activation time was changed in the range from 1 to 5 s. The concentration of Sunset Yellow was 5 × 10^−8^ mol L^−1^ of Sunset Yellow. The obtained results are presented in Figure 4B. Based on the obtained results, an activation time of 2 s was chosen for further studies as, under these conditions, the highest peak current of Sunset Yellow was observed.

### 2.4. Optimization of Sunset Yellow Accumulation Conditions

**Figure 5:**
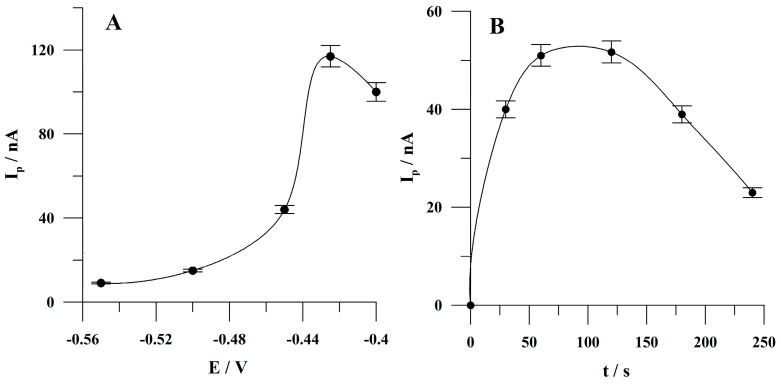
The impact of accumulation potential (**A**) and accumulation time (**B**) on Sunset Yellow peak current. The concentration of Sunset Yellow was 5 × 10^−8^ mol L^−1^ (**A**) and 2 × 10^−8^ mol L^−1^ (**B**). Accumulation time: 60 s (**A**); accumulation potential: −0.425 (**B**). The error bars represent the standard deviation (n = 3).

The impact of accumulation potential on the peak current of Sunset Yellow was studied in the range from −0.4 to −0.55 V. The accumulation time was 60 s. The concentration of Sunset Yellow was 5 × 10^−8^ mol L^−1^. The results obtained during the study are shown in Figure 5A and indicate that the peak height of Sunset Yellow increased extremely as the accumulation potential was changed from −0.4 to −0.425 V and then decreased at more negative potential values. Further research was performed at the chosen accumulation potential of −0.425 V.

The effect of accumulation time on the peak height of Sunset Yellow was studied from 30 to 180 s. The Sunset Yellow concentration was 2 × 10^−8^ mol L^−1^. The obtained research results are shown in Figure 5B. It was observed that Sunset Yellow peak current increased to 60 s and then decreased weakly at a longer preconcentration time. The probable reason for the decrease in the Sunset Yellow peak height at a longer accumulation time is a saturation of the surface of the microelectrode array with the molecules of the analyte. This effect makes electron exchange between the analyte and the working electrode’s surface more difficult. Further research was conducted at 60 s of accumulation.

### 2.5. Calibration Studies

**Figure 6:**
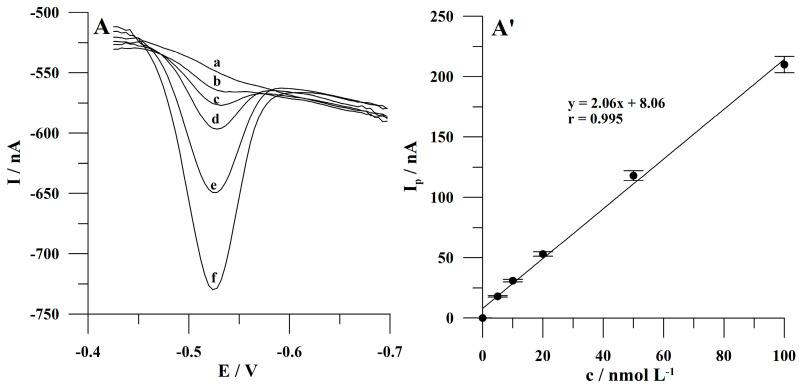
Adsorptive stripping voltammograms obtained for increasing concentration of Sunset Yellow (**A**) and the corresponding linear calibration graph (**A’**). Sunset Yellow concentration: (a) 0; (b) 5 × 10^−9^; (c) 1 × 10^−8^; (d) 2 × 10^−8^; (e) 5 × 10^−8^; (f) 1 × 10^−7^ mol L^−1^. Accumulation conditions: −0.425 V, 60 s. The error bars represent the standard deviation (n = 3).

After the optimization of the main measurement conditions, calibration studies were performed. The calibration plot for an accumulation time of 60 s was a straight line from 5 × 10^−9^ to 1 × 10^−7^ mol L^−1^ of Sunset Yellow and obeyed the equation y = 2.06× + 8.06, where y and x are the peak current (expressed in nA) and the Sunset Yellow concentration (expressed in nmol L^−1^), respectively. The linear correlation coefficient r was 0.995. The relative standard deviation for the determination of Sunset Yellow at a concentration of 2 × 10^−8^ mol L^−1^ was 4.1% (n = 7). The limit of detection, estimated as three times the standard deviation of the intercept divided by the slope of the calibration graph, was 2.1 × 10^−9^ mol L^−1^ (accumulation time 60 s). Voltammograms obtained for increasing concentrations of Sunset Yellow are presented in Figure 6A. The corresponding calibration graph is shown in Figure 6A’.

### 2.6. Repeatability and Reproducibility Studies

To examine the repeatability of the results obtained using a solid bismuth microelectrode array, the analytical signal of Sunset Yellow was recorded seven times in a sequential manner, one by one, for a solution containing Sunset Yellow at a concentration of 2 × 10^−8^ mol L^−1^. The obtained RSD value was equal to 4.1%.

The reproducibility of the obtained results was also estimated based on measurements conducted from a solution containing Sunset Yellow at a concentration of 5 × 10^−8^ mol L^−1^ within five consecutive days. The obtained RSD value was equal to 4.7%.

Based on these results, it can be concluded that the presented procedure is characterized by satisfactory precision.

### 2.7. Interference Studies

The potential interference effects that may be observed during the analysis of the beverages and environmental water samples, including inorganic ions and organic substances, were studied using a solution containing Sunset Yellow at a concentration of 5 × 10^−8^ mol L^−1^. The accumulation conditions were −0.425 V and 60 s. The obtained results are presented in Table 1. The study revealed that the majority of the examined inorganic ions and organic substances did not exhibit a substantial impact on the peak current of Sunset Yellow, even at a high excess. The occurrence of potential interferences was also investigated in the presence of two other example azo dyes, amaranth and tartrazine. Due to the relatively low content of these dyes in the real samples, the same concentration as that of Sunset Yellow was tested. The study revealed that the presence of amaranth did not significantly affect the Sunset Yellow peak current. However, tartrazine was demonstrated to exert a considerable effect on the Sunset Yellow analytical signal, resulting in its partial attenuation. This phenomenon is attributed to the comparable peak potentials exhibited by these azo dyes. The results presented herein indicate that the developed analytical procedure was found to be satisfactory in terms of its selectivity.

### 2.8. Analytical Application

**Figure 7:**
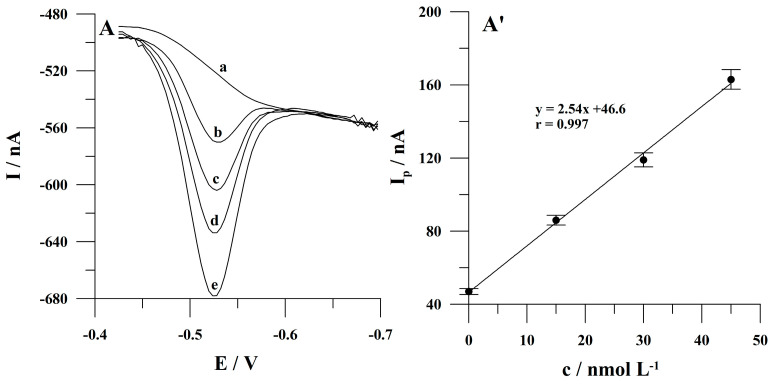
Adsorptive stripping voltammograms obtained during analysis of the isotonic beverage sample (**A**): (a) supporting electrolyte; (b) supporting electrolyte with isotonic beverage sample; (c) as (b) + 1.5 × 10^−8^ mol L^−1^; (d) as (c) + 1.5 × 10^−8^ mol L^−1^; (e) as (d) + 1.5 × 10^−8^ mol L^−1^ of Sunset Yellow. Accumulation at −0.425 V within 60 s. (**A’**) The corresponding standard addition graph. The error bars represent the standard deviation (n = 3).

The developed voltammetric procedure of Sunset Yellow determination was employed for the analysis of an isotonic beverage. A sample of a volume of 5 μL (factor of dilution: 2000) was added to the voltammetric cell containing the supporting electrolyte. The beverage sample preparation is described in the Preparation of Beverages and Water Samples Section. The voltammetric analysis was carried out using the standard addition method (accumulation time of 60 s). The voltammograms obtained during the isotonic beverage analysis and the corresponding standard addition graph are presented in Figure 7A and A’, respectively. The obtained analysis result was 16.6 mg L^−1^ with a standard deviation of 4.8% (n = 5). This result was consistent with the results of 16.4 mg L^−1^ obtained by high-performance liquid chromatography used as a comparative method. In consideration of the presented results, it can be concluded that the elaborated voltammetric procedure of Sunset Yellow determination using the solid bismuth microelectrode array can be applied for the analysis of isotonic beverages.

**Figure 8:**
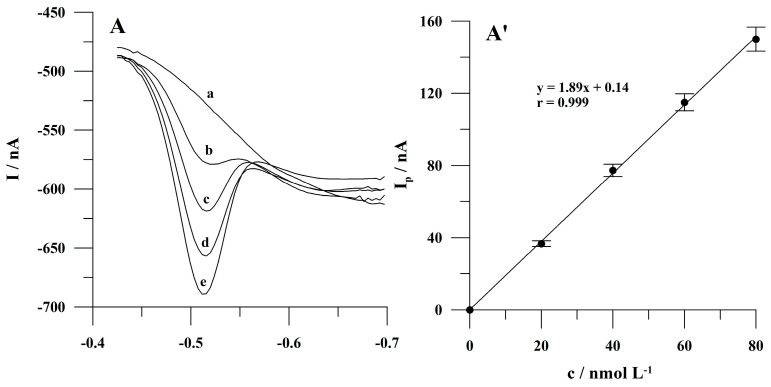
Adsorptive stripping voltammograms obtained during analysis of a real water sample (**A**): (a) diluted water sample; (b) as (a) + 2 × 10^−8^ mol L^−1^; (c) as (b) + 2 × 10^−8^ mol L^−1^; (d) as (c) + 2 × 10^−8^ mol L^−1^; (e) as (d) + 2 × 10^−8^ mol L^−1^ of Sunset Yellow. Accumulation at −0.425 V within 60 s. (**A’**) The corresponding standard addition graph. The error bars represent the standard deviation (n = 3).

A practical application of the developed voltammetric procedure was also examined by conducting recovery studies from the environmental water samples collected from the Bystrzyca River (a river located in the eastern regions of Poland) that was spiked with a fixed concentration of Sunset Yellow. The dilution factor of the natural water sample was equal to 2. A natural water sample was analyzed using the standard addition method (accumulation time was 60 s). The obtained voltammograms are shown in Figure 8A. The corresponding standard addition graph is presented in Figure 8A’. The average recovery values were in the range from 96.5 to 102% with a relative standard deviation (RSD) of 4.1%. The obtained results confirmed the applicability of the developed analytical procedure for the determination of Sunset Yellow in real water samples.

## 3. Materials and Methods

### 3.1. Instrumentation

The research was conducted using a μAutolab analyzer (from ECO/Chemie, Utrecht, The Netherlands). A traditional three-electrode system was used, with the solid bismuth microelectrode array as a working electrode, an Ag/AgCl/NaCl reference electrode, and a platinum wire counter electrode. The volume of the voltammetric cell was 10 mL. The working electrode was constructed as described in detail in [45] in the course of the fabrication of a gold microelectrode array. Shortly, for the construction of the array of solid bismuth microelectrodes, a homemade silica preform containing about 169 holes, made in the Laboratory of Optical Fibers Technology at our university, was utilized. The outer diameter of the preform was equal to 3 mm. The holes have a circular shape with a diameter of about 19 μm. The minimal distance between holes was 130 μm. The holes in the preform were filled with melted bismuth using a procedure similar to that reported in [46,47]. Metallic bismuth was melted at a temperature of about 400 °C and then was pressed in the holes of the preform under a pressure of about 20 bars. Then, 6 mm of the preform filled with bismuth was placed in the PEEK housing of a diameter of 6 mm. An electrical contact from the solid bismuth microelectrode array was prepared using graphitized carbon black powder and a copper wire. In this way, an eco-friendly, durable, and long-term-use working microelectrode array was fabricated. The surface of the working solid bismuth microelectrode array was polished every day directly before the voltammetric measurements using sandpaper of 1500 and 2500 grit and then cleaned with deionized water in an ultrasonic bath in 30 s. A real image of the solid bismuth microelectrode array was taken using an MA200 Inverted Metallographic Microscope Nikon (Tokyo, Japan).

### 3.2. Reagents

Ammonia buffer (pH 9.7) at 1 mol L^−1^ was prepared using HCl and NH_4_OH; Suprapur reagents were bought from Merck (Rahway, NJ, USA). The Sunset Yellow reagent was purchased from Sigma-Aldrich (St. Louis, MO, USA). A standard solution of Sunset Yellow at a concentration of 5 × 10^−3^ mol L^−1^ was prepared by dissolution of the reagent in deionized water and stored in a refrigerator. A working solution of Sunset Yellow at a concentration of 1 × 10^−5^ mol L^−1^ was prepared daily by an appropriate dilution of a standard solution in deionized water. The reagents used to examine interference effects were obtained from Sigma. The isotonic beverage was bought in a local market. All chemicals used in the described research were of analytical reagent grade or Suprapur. Deionized water obtained from a Milli-Q system purification was used for preparing all solutions.

### 3.3. Preparation of Beverages and Water Samples

A real water sample taken from the Bystrzyca River (Poland) and the isotonic beverage were stored in a refrigerator until use. Prior to analysis, the environmental water sample and the beverage sample were filtered using a membrane filter (0.45 μm) and then added to an electrochemical cell.

### 3.4. Standard Procedure of Measurement

The analyzed sample was pipetted into the voltammetric cell. Then, 1 mL 1 mol L^−1^ of ammonia buffer (pH 9.7) was added and made up to a volume of 10 mL with deionized water. The adsorptive stripping voltammetric measurements were performed by applying the following sequence of potential values to the solid bismuth microelectrode array:

−2.75 V within 2 s: During this so-called activation stage, the bismuth oxides that could form on the surface of the microelectrode as a result of contact between the electrode material and the oxygen dissolved in the solution underwent a reduction process whereby they were transformed into a metallic form. Moreover, during this step, organic species remained on the surface of the working electrode after the previous measurement underwent a desorption process and were transferred to the bulk of the solution.
−0.425 V within 60 s: During this step, Sunset Yellow accumulated on the surface of the solid bismuth microelectrode array;
3. After a rest ten-second equilibration period, a square wave voltammogram was registered; the potential was changed from −0.4 to −0.7 V. The square wave parameters were as follows: frequency 100 Hz, amplitude 50 mV and step potential 4 mV.

The research was conducted without the deoxygenation of the solutions.

### 3.5. Methodology of Chromatographic Measurements

The analysis of Sunset Yellow was performed using reversed-phase high-performance liquid chromatography in conformance with the method described in [48]. The studies were performed with the use of a Varian (Palo Alto, CA, USA) HPLC system equipped with a diode-array detector (DAD, type 335), high-pressure gradient systems composed of two pumps (type 210), and an autosampler module (type 410) with a column thermostat. Separation was obtained with a chromatographic column Gemini C18 (150 × 4.6 mm; 3 µm), connected with a pre-column Gemini C18 4 × 3 mm (Phenomenex, Torrance, CA, USA) using 0.02 mol/L aqueous solution of ammonium acetate with a pH of 4.5 adjusted by adding acetic acid (solvent A) and methanol (solvent B) at a constant flow rate of 0.6 mL/min. The gradient (*v*/*v*) was generated, decreasing from 85% of solvent A to 20% in 6 min, followed by increasing to 65% in 10 min and to 85% in 15 min. Before each injection, the system was stabilized for 5 min with the initial A/B ratio (85:15, *v*/*v*). Before use, the mobile phase was filtered through a 0.45 µm micro-pore filter membrane. The injection volume was 20 µL. Chromatograms were recorded at 484 nm and a column temperature of 30 °C. The concentration of Sunset Yellow in the sample was determined from the calibration curve equation plotted based on the analysis of standard solutions. The identification of Sunset Yellow was performed based on the retention time and the UV spectrum of the reference substance.

## 4. Conclusions

This article reports for the first time the application of a novel working electrode for stripping voltammetric measurements—a long-term-use solid bismuth microelectrode array. The construction method employed in the fabrication of the presented microelectrode ensures the attainment of a sensor that is both stable and durable, and which is suitable for long-term use. Additionally, it was observed that the recorded currents were enhanced and more resistant to interference in comparison to a single solid bismuth microelectrode. The proposed working microelectrode array was applied for the development of an analytical procedure for the determination of Sunset Yellow FCF by adsorptive stripping voltammetry in isotonic beverages and environmental water samples. The described voltammetric procedure is characterized by a low detection limit equal to 1.7 × 10^−9^ mol L^−1^ (for an accumulation time of 60 s). Interference studies confirmed the high selectivity of the developed analytical procedure. The correctness of the presented procedure was confirmed by the compatibility of the results of the analysis of the isotonic beverage sample and that obtained by high-performance liquid chromatography as a comparative method. Furthermore, satisfactory recovery values were obtained during the analysis of spiked environmental water samples.

## Figures and Tables

**Table 1:** Relative analytical signal of Sunset Yellow with and without the presence of interferents. The concentration of Sunset Yellow was 5 × 10^−8^ mol L^−1^.

Interfering Ion/Substance	A Molar Excess of Interfering Ion/Substance	^1^ Relative Signal of Sunset Yellow ± SD
K^+^	500	0.92 ± 0.042
Na^+^	500	0.93 ± 0.045
Ca^2+^	500	0.94 ± 0.036
Mg^2+^	500	1.01 ± 0.041
Fe^3+^	100	0.93 ± 0.037
NO^3−^	1000	0.97 ± 0.043
NO^2−^	1000	0.95 ± 0.044
Cl^−^	500	0.96 ± 0.039
Ascorbic acid	500	0.99 ± 0.043
Vitamin B_1_	100	0.98 ± 0.045
Vitamin B_6_	100	1.05 ± 0.039
Glucose	1000	0.90 ± 0.047
Amaranth	1	1.03 ± 0.049
Tartrazine	1	0.38 ± 0.045

^1^ Relative signal—peak current ratio in the presence and absence of a molar excess of the interferent.

## Data Availability

Data are contained within the article.
